# Insulin down-regulates the expression of ubiquitin E3 ligases partially by inhibiting the activity and expression of AMP-activated protein kinase in L6 myotubes

**DOI:** 10.1042/BSR20150017

**Published:** 2015-08-20

**Authors:** Hu-Ping Deng, Jia-Ke Chai, Chuan-An Shen, Xi-Bo Zhang, Li Ma, Tian-Jun Sun, Qing-Gang Hu, Yun-Fei Chi, Ning Dong

**Affiliations:** * Burns Institute, Department of Burn and Plastic Surgery, Burn and Plastic Surgery Hospital, First Affiliated Hospital of General Hospital of PLA, Beijing 100048, China

**Keywords:** AMP-activated protein kinase (AMPK), insulin, muscle atrophy F-box (MAFbx), muscle RING finger 1 (MuRF1), myotubes

## Abstract

While insulin is an anabolic hormone, AMP-activated protein kinase (AMPK) is not only a key energy regulator, but it can also control substrate metabolism directly by inducing skeletal muscle protein degradation. The hypothesis of the present study was that insulin inhibits AMPK and thus down-regulates the expression of the ubiquitin E3 ligases, muscle atrophy F-box (MAFbx) and muscle RING finger 1 (MuRF1) in skeletal muscle cells. Differentiated L6 myotubes were treated with 5-aminoimidazole-4-carboxamide-1-β-4-ribofuranoside (AICAR) and/or compound C to stimulate and/or block AMPK respectively. These treatments were also conducted in the presence or absence of insulin and the cells were analysed by western blot and quantitative real-time PCR. In addition, nuleotide levels were determined using HPLC. The activation of AMPK with AICAR enhanced the mRNA levels of MAFbx and MuRF1. Insulin reduced the phosphorylation and activity AMPK, which was accompanied by reduced MAFbx and MuRF1 mRNA levels. Using a protein kinase B (PKB/Akt) inhibitor, we found that insulin regulates AMPK through the activation of Akt. Furthermore, insulin down-regulated AMPK α2 mRNA. We conclude that insulin inhibits AMPK through Akt phosphorylation in L6 myotubes, which may serve as a possible signalling pathway for the down-regulation of protein degradation. In addition, decreased expression of AMPK α2 may partially participate in inhibiting the activity of AMPK.

## Abstract (precis)

We conclude that insulin inhibits AMPK through Akt phosphorylation in L6 myotubes, which may serve as a possible signalling pathway for the down-regulation of protein degradation. Besides, decreased expression of AMPK α2 may partially participate in inhibiting the activity of AMPK.

## INTRODUCTION

Insulin is a main anabolic hormone involved in the regulation of glucose, protein and lipid metabolisms [1–3]. In some critical illnesses, intensive insulin therapy is utilized to combat the harmful effects of hyperglycaemia. Most of the effects of insulin depend on its ability to activate the phosphatidylinositol 3-kinase (PI3K)/Akt pathway, subsequently phosphorylate glycogen synthase kinase 3-β (GSK3-β), tuberous sclerosis protein-2 (TSC2), bcl-2 antagonist of cell death (BAD), forkhead box O (Foxo) etc. [1–3]. These Akt targets are important for the regulation of protein metabolism, inhibition of lipid lysis, promotion of fatty acid synthesis and glycogen synthesis etc. [1].

ATP is essential in the above processes and serves as an energy supply whereas AMPK (AMP-activated protein kinase) is a key regulator of energy metabolism. AMPK is comprised of three subunits (α, β and γ), among which the α subunit (including the α1 and α2 sub-types) is the catalytic subunit [4]. AMPK is regulated by the energy status of the internal environment (AMP–ATP ratio). Elevated AMP–ATP ratios makes AMPK vulnerable to phosphorylation at Thr^172^ and activation by several upstream kinases, such as serine/threonine kinase 11 (LKB1) or calcium/calmodulin-dependent protein kinase kinase (CaMKK) [5]. Activated AMPK down-regulates anabolism, facilitating a decrease in ATP expenditure. However, activated AMPK induces catabolism of glucose, lipids and proteins and thus, enhances ATP synthesis [6]. Activated AMPK helps cells produce more ATP for use in metabolism. Meanwhile, AMPK activation induces an up-regulation of catabolism and a down-regulation of anabolism [7]. All of the properties of AMPK mentioned above suggest that it might interact in insulin signalling pathways.

Recent evidence suggests that AMPK plays a role in the insulin signalling pathway. Activated AMPK inhibits mammalian target of rapamycin (mTOR) by phosphorylating and activating TSC2, which in turn regulates the protein synthesis modulating molecules 70 kDa ribosomal protein S6 kinase (p70S6k) [8] and elongation factor 2 (eEF2) [9] and subsequently, reduces protein synthesis. The question remains whether insulin regulates AMPK activity. In myocardial cells, the activity of AMPK can be inhibited by insulin [10]. As a key target of insulin, skeletal muscle is the main location of protein metabolism. Furthermore, protein degradation can be inhibited by insulin in various conditions *in vivo* and *in vitro* and its main mechanism is the activation of Akt and the inhibition of Foxo, which in turn decreases the expression of the Ubiquitin E3 ligases muscle atrophy F-box (MAFbx) and muscle RING finger 1 (MuRF1) (reference). The mechanism of how insulin regulates AMPK in skeletal muscle is still unclear. Therefore, the aim of the present study was to identify the effect of insulin on AMPK and its role in regulating protein breakdown in L6 myotubes. The ultimate goal of the present study is to lay the foundation for the investigation of clinical insulin application for the treatment of the critically ill, diabetics and other insulin-related clinical problems.

## MATERIALS AND METHODS

### Cell culture and treatment

L6 rat skeletal muscle cells were obtained from Peking Union Medical College and maintained in DMEM (Dulbecco's Modified Eagle Medium; 4.5 g/l glucose, Hyclone) with 10% FBS, 100 units/ml penicillin and 100 μg/ml streptomycin at 37°C in 5% CO_2_. To differentiate L6 myoblasts into myotubes, the culture medium was substituted with DMEM plus 2% horse serum after the L6 myoblast cells became confluent. Four days after the initiation of the differentiation, the cells were treated with 10 μM cytosine arabinoside (Sigma) for 24 h to remove the dividing myoblasts. Prior to being cultured with any stimulators or inhibitors, the myotubes were serum starved for 12 h. L6 myotubes were treated with 1 mM 5-aminoimidazole-4-carboxamide-1-β-4-ribofuranoside (AICAR, Sigma) and (or) 100 nM insulin (Sigma) for 30 min for the analysis of phosphorylation levels of proteins, 1 h for the analysis of nt or 24 h for mRNA analysis. In some experiments, L6 myotubes were pre-treated with 1 mM AICAR, 20 μM compound C (AMPK inhibitor, Sigma, St. Louis, MO) or Akt inhibitor IV (Akt inhibitor, Sigma) for 1 h and the treatments continued through the duration of the experiments.

### Protein extraction and western blotting

Protein was extracted from L6 myotubes with RIPA (radioimmunoprecipitation assay) buffer, containing 50 mM Tris/HCl (pH 7.4), 150 mM NaCl, 1% NP-40, 0.25% Na-deoxycholate, 1 mM EDTA, 1% protease inhibitors cocktail (Pierce), 1 mM PMSF (Pierce), 1 mM Na_3_VO_4_ (Sigma) and 50 mM NaF (Sigma). Protein concentration was quantified with a BCA Protein Assay Kit (Pierce) according to the manufacturer's instructions.

Before the samples were loaded on to the SDS/PAGE gels, 40 μg of total protein was mixed with loading buffer and denatured for 5 min at 100°C in a water bath. The protein was transferred to PVDF immuno-blotting membranes using a Electrophoretic Transfer Cell (Mini Trans-Blot®, Bio-Rad) after being separated by electrophoresis in a Tetra Cell (Mini-PROTEIN®, Bio-Rad). Subsequently, the PVDF blots were incubated in blocking buffer (5% dry fat-free milk in TBST) for 30 min followed by incubation for 1.5 h at room temperature in blocking buffer containing the appropriate dilution of primary antibody [anti-phosphor-AMPKα (Cell Signaling), anti-phosphor-ACC at (Cell Signaling), anti-AMPKα (Cell Signaling)]. The membranes were washed (four times at 5 min/wash) in TBST and then incubated for 1 h in blocking buffer containing the appropriate horseradish peroxidase (HRP)-conjugated secondary antibody (Zhong Shan Golden Bridge Biotechnology). After washing, as described above, the blots were incubated in a chemiluminescent substrate solution (Pierce) for the desired amount of time to visualize the bands. Equal protein loading was determined by stripping each membrane and re-probing for GAPDH (glyceraldehyde-3-phosphate dehydrogenase, Zhong Shan Golden Bridge Biotechnology).

### Total RNA extraction and quantitative real time PCR

The total RNA from was extracted from the treated L6 myotubes with TRIZOL reagents (Invitrogen) according to the manufacturer's instructions and quantified with ultraviolet spectrophotometry (Beckman). cDNA was synthesized with Reverse Transcriptase M-MLV (RNase H^−^; Takara).

The measurement of relative RNA levels was performed according to methods described previously [11]. Briefly, 1 μl of a 1:25 diluted cDNA template was mixed with 10 μl of Fast SYBR Green Master Mix (Applied Biosystems), 7 μl of distilled H_2_O, 2 μl of each forward and reverse primers (synthesized by SBS Genetech, Table 1) to an ultimate concentration of 300 nM. Real-Time PCR System (Applied Biosystems) was used for amplification and the cycling conditions were: denature at 95°C for 20 s followed by 40 cycles of annealing at 95°C for 3 s and extension at 60°C for 30 s). Amplification plots were used to determine the ‘cycle threshold (Ct)’ values. *GAPDH* was used as the endogenous control. Samples were analysed in triplicate. The fold change in expression was then obtained in RQ value.

**Table 1:** Primers for gene transcripts

Genes transcripts	Forward primer (5'–3')	Reverse primer(5'–3')
AMPK α1	ATC CGC AGA GAG ATC CAG AA	CGT CGA CTC TCC TTT TCG TC
AMPK α2	GCT GTG GAT CGC CAA ATT AT	GCA TCA GCA GAG TGG CAA TA
MAFbx [11]	CCA TCA GGA GAA GTG GAT CTA TGT T	GCT TCC CCC AAA GTG CAG TA
MuRF1 [11]	TGA CCA AGG AAA ACA GCC ACC AG	CTC ACT CTT CTT CTC GTC CAG GAT GG
*GAPDH* [11]	TGC ACC ACC AAC TGC TTA	GGA TGC AGG GAT GAT GTT C

### AMP/ADP/ATP determination

Myotubes were washed twice in ice-cold PBS, the nts were extracted from the L6 myotubes by scrapping in ice-cold 5% perchloric acid. The extracts were incubated on ice for 30 min and then vortexed and neutralized with 2 M potassium bicarbonate and subsequently centrifuged at 13000 ***g*** for 8 min at 4°C. The supernatants were collected and frozen at −70°C.

Determination of nt levels were carried out by HPLC analysis, as described previously [12]. A 20 μl of sample was injected into the HPLC system (1200 Series Quaternary LC System, Agilent Technology) with 0.2 M phosphate buffer (0.1 M Na_2_HPO_4_, 0.1 M KH_2_PO_4_, 5 mM tetrabutyl ammonium bromide, pH 6.25) plus 10% methanol. The nts were analysed, eluted at a flow rate of 1.3 ml/min and detected spectrophotometrically at 254 nm. The order of eluted nts was ATP, ADP and AMP. The total retention time was within 6 min and the gradient was run for 10 min to ensure full separation. By comparing the retention times of the samples to standards, the identification of ATP, ADP and AMP was made, whereas the concentrations were determined using the external standard method. Briefly, different concentrations (0, 2, 10, 20, 40, 80, 160, 320 μM) of each standards were determined and expressed in the formula as y=ax + b (y represents area, whereas x represents concentration). Finally, the AMP–ATP ratio and ADP–ATP ratio were obtained. Energy charge (EC) was calculated by the following formula: EC=(1/2ADP + ATP)/(ATP + ADP + AMP).

### Statistical analysis

Results are presented as the mean ± S.D. A Student's *t*test was used for statistical analyses. Differences were considered significant at a *P*-value less than 0.05.

## RESULTS

### AICAR induces the phosphorylation of AMPK and increases the mRNA levels of *MAFbx* and *MuRF-1*

Previously, we found that at 1 mM or higher AICAR significantly stimulated the phosphorylation of AMPKα at Thr^172^ and the maximum phosphorylation of AMPK was attained 30 min after the addition of 1 mM AICAR (result not shown). Thereafter, in the present study, AICAR was used at 1 mM for 30 min. Additionally, acetyl CoA carboxylase (ACC), a target of AMPK, shows an increase in phosphorylation after AICAR treatment. Pre-treatment with compound C (20μM), an AMK inhibitor, blocked AICAR-induced phosphorylation of AMPK (Figure. 1A).

**Figure 1:**
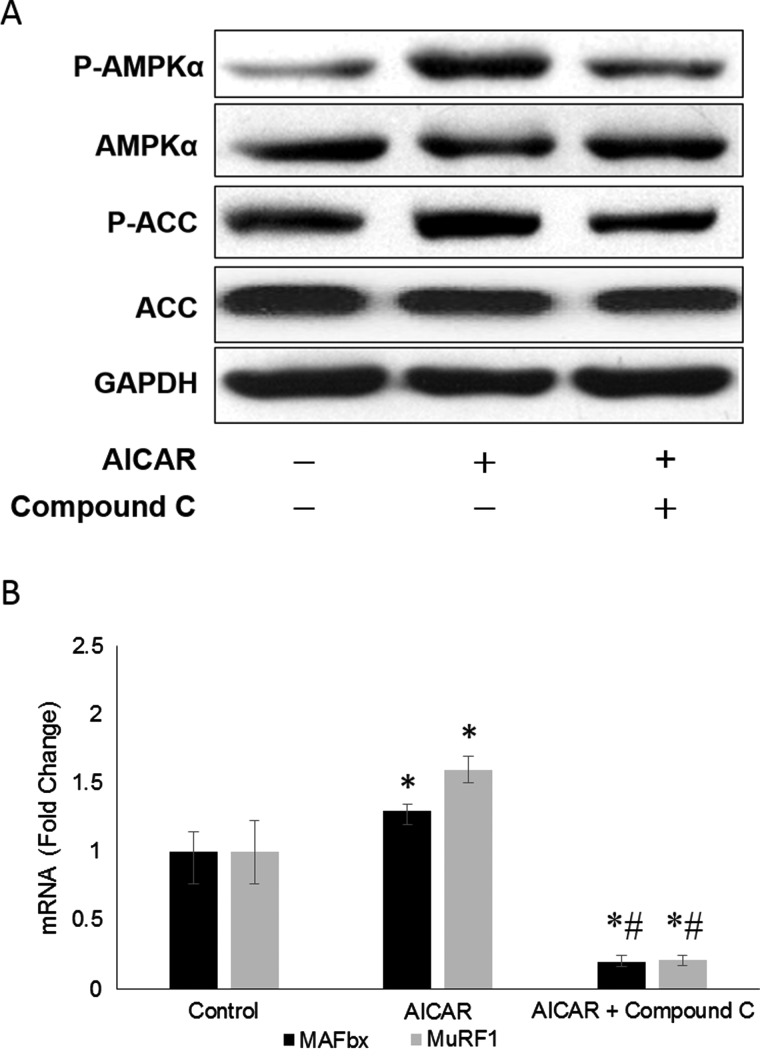
Effects of AICAR and compound C on p-AMPK, p-ACC, MAFbx and MuRF1 Effects of AICAR and compound C (CC) on p-AMPK, p-ACC (**A**) and mRNA expression of MAFbx and MuRF1 (**B**) in L6 myotubes. L6 myotubes were pre-treated with 20 μM CC or without CC for 1 h, followed by treatment with AICAR (1 mM) for 30 min (**A**) or 24 h (**B**). (**A**) After stimulation with AICAR for 30 min, p-AMPK at Thr^172^ and p-ACC at Ser^79^ were measured by western blotting, with GAPDH as the internal standard. Shown are representative blots from three independent experiments. (**B**) At 24 h after AICAR treatment, extracted RNA from L6 myotubes were assayed with qRT-PCR. Data were normalized to *GAPDH* and the values for the control group set at 1.0. Data are expressed as means ± S.D. (*n*=3). *P<0.05 compared with control. #*P*<0.05 compared with AICAR treatment group.

Next, we examined the effect of the activation of AMPK by AICAR on the mRNA levels of MAFbx and MuRF1. At 24 h, the mRNA levels of MAFbx and MuRF1 increased in the AICAR treatment group, whereas pre-treatment with compound C in AICAR-induced cells not just prevented, but indeed significantly impaired the expression of MuRF1 and MAFbx mRNAs as compared with the control group. (Figure 1B).

### Insulin reduces the phosphorylation of AMPK and inhibits its activity

To assess the effect of insulin treatment on the phosphorylation state and activity of AMPK, L6 myotubes were treated with insulin (100 nM) for 30 min, followed by western blot analyses of phosphor-AMPK (p-AMPKα) and phosphor-ACC (p-ACC). Insulin markedly reduced the phosphorylation of both AMPKα at Thr^172^ and ACC at Ser^79^. When pre-treated with AICAR for 1 h before insulin treatment, p-AMPK and p-ACC did not change compared with control. Pre-treatment with compound C prior to insulin treatment reduced p-AMPK and p-ACC compared with control, which was similar to treatment with insulin alone (Figure 2A). As shown in Figure 1, AICAR increased both p-AMPK and p-ACC. Thus, it could be deduced that insulin inhibited AICAR-induced AMPK phosphorylation and activity in L6 myotubes.

**Figure 2:**
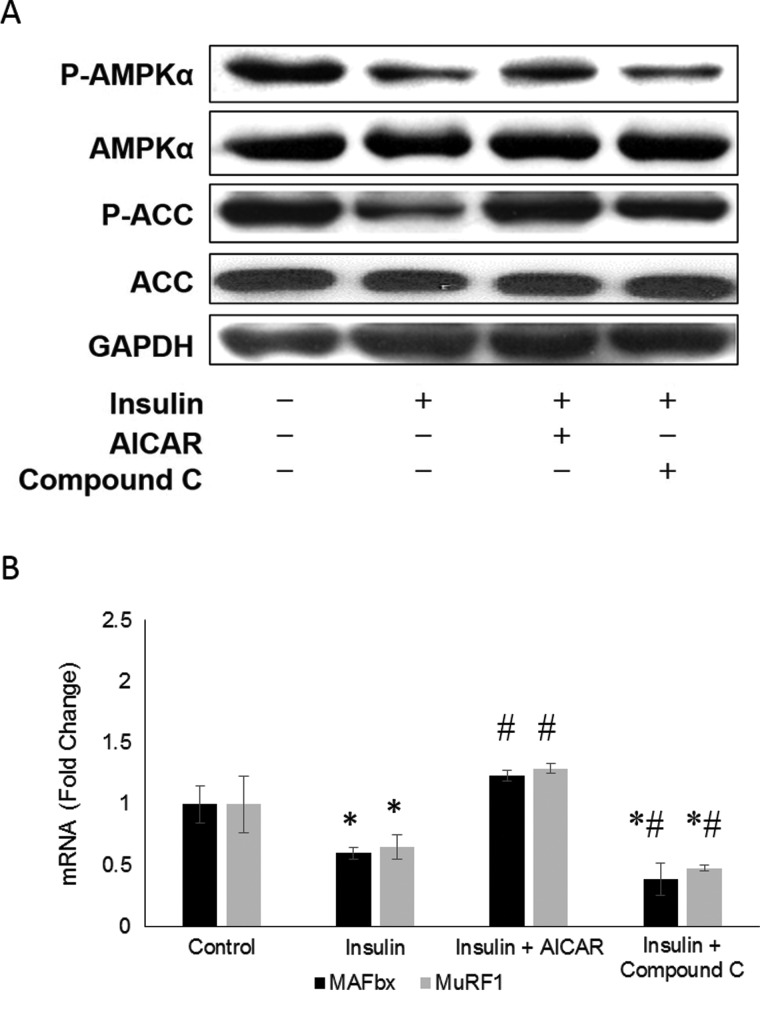
Effects of insulin on p-AMPK, p-ACC, MAFbx and MuRF1 Effects of insulin on p-AMPK, p-ACC (**A**) and mRNA expression of MAFbx and MuRF1 (**B**) in L6 myotubes. L6 myotubes were pre-treated with AICAR (1 mM), compound C (CC; 20 μM) or not with AICAR and CC for 1 h, followed by addition of insulin (100 nM) for 30 min (**A**) or 24 h (**B**). (**A**) At 30 min, p-AMPK and p-ACC were measured by western blotting, with GAPDH as the internal standard. Shown are representative blots from three independent experiments. (B) At 24 h, extracted RNA from L6 myotubes were assayed with qRT-PCR. Data were normalized to *GAPDH* and the values for the control group set at 1.0. Data are expressed as means ± S.D. (*n*=3). *P<0.05 compared with control. #*P*<0.05 compared with insulin only treatment group.

Because insulin is an anabolic hormone that inhibits muscle protein degradation, mRNA expression of MAFbx and MuRF1 were determined 24 h after insulin treatment. The mRNA expression of MAFbx and MuRF1 were significantly lower than the control after insulin treatment (Figure 2B). Pre-treatment with AICAR prior to insulin stimulation prevented this reduction and resulted in a statistically significant increase compared with the insulin treatment group in MAFbx and MuRF1 mRNA (Figure 2B). In contrast, when myotubes were pre-treated with compound C prior to the addition of insulin, the mRNA expression of both MAFbx and MuRF1 was significantly reduced (Figure 2B).

### Insulin reduces the phosphorylation of AMPK and inhibits its activity through the activation of AKT

Many of the downstream cellular functions induced by insulin utilize the activation of Akt to promote signal transduction. To investigate whether the effects of insulin on AMPK phosphorylation and activity occur through Akt signalling, we used an Akt inhibitor (Akt Inhibitor IV) as the pre-treatment agent. Consistent with Figure 2(A), insulin reduces p-AMPK and p-ACC. Pre-treatment with the Akt inhibitor prevented the reduction in p-AMPK and p-ACC induced by insulin (Figure 3). These findings suggest that Akt is required for insulin to inhibit AMPK.

**Figure 3:**
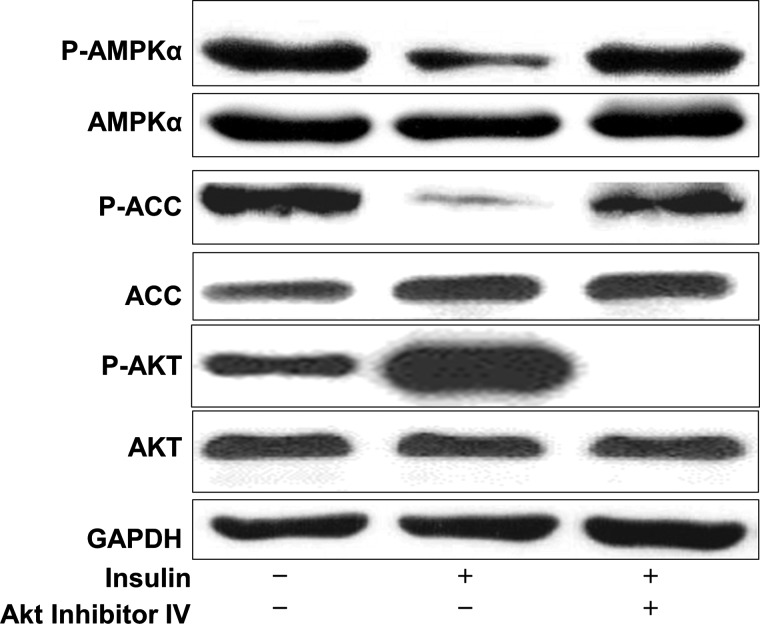
Effect of Akt inhibitor on inhibition of p-AMPK and p-ACC by insulin The effect of Akt inhibitor (Akt inhibitor IV) on inhibition of p-AMPK and p-ACC by insulin in L6 myotubes. L6 myotubes were pre-treated with Akt inhibitor IV (1 μM) for 1 h, followed by addition of insulin (100 nM) or without insulin for 30 min. p-AMPK, p-ACC and phosphor-Akt (p-Akt) were measured by western blotting, with GAPDH as the internal standard. Shown are representative blots from three independent experiments.

### Insulin down-regulates steady state expression of AMPK α2 mRNA

To understand more about the mechanism of how insulin reduces AMPK activity, we determined the mRNA levels of AMPK α1 and AMPK α2 subunits in myotubes in the presence of insulin. Insulin reduces AMPK α2 mRNA levels. Pre-treatment with AICAR did not change this reduction, but pre-treating the cells with compound C prior to insulin treatment further potentiated this reduction. Insulin did not affect AMPK α1 mRNA expression and the AICAR or compound C treatments did not change this result (Figure 4).

**Figure 4:**
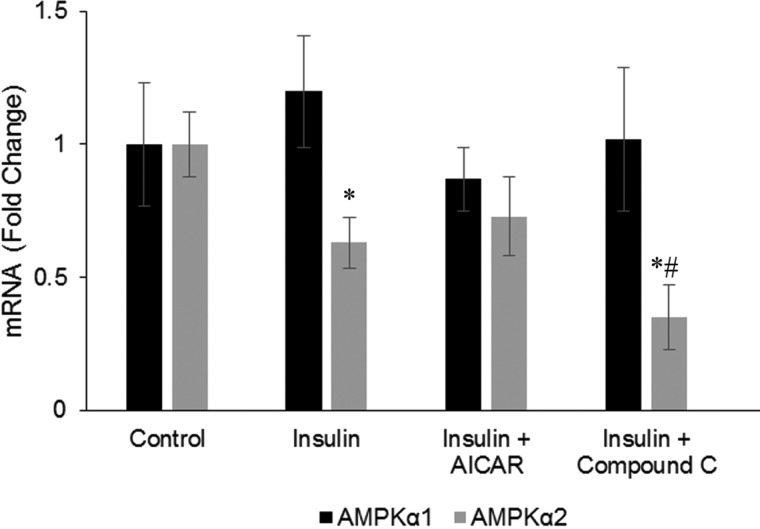
Effect of insulin on AMPK α1 and α2 Effects of insulin on mRNA expression of AMPK α1 and α2. L6 myotubes were either untreated (control) or pre-treated with 1 mM AICAR or 20 μM CC or not with AICAR and CC for 1 h, followed by stimulation with 100 nM insulin for 24 h. Extracted RNA from L6 myotubes were assayed with qRT-PCR. Data were normalized to *GAPDH* and the values for the control group set at 1.0. Values are expressed as means ± S.D. (*n*=3). *P<0.05 compared with control. #*P*<0.05 compared with insulin only group.

### Insulin has no effects on AMP–ATP ratio, ADP–ATP ratio and energy charge

The AMP–ATP ratio is a key factor involved in regulating the phosphorylation of AMPK. Thus, we determined the AMP–ATP ratio, the ADP–ATP ratio and the EC. By using HPLC methods, the concentrations of AMP, ADP and ATP were determined, followed by a calculation of AMP–ATP, ADP–ATP and EC. There were no differences for both the ratios and the EC between the insulin treatment group and the control group (*P*>0.05; Figure 5). These data suggest that the reduction in AMPK phosphorylation by insulin is not because of AMP–ATP or ADP–ATP ratio differences or energy changes.

**Figure 5:**
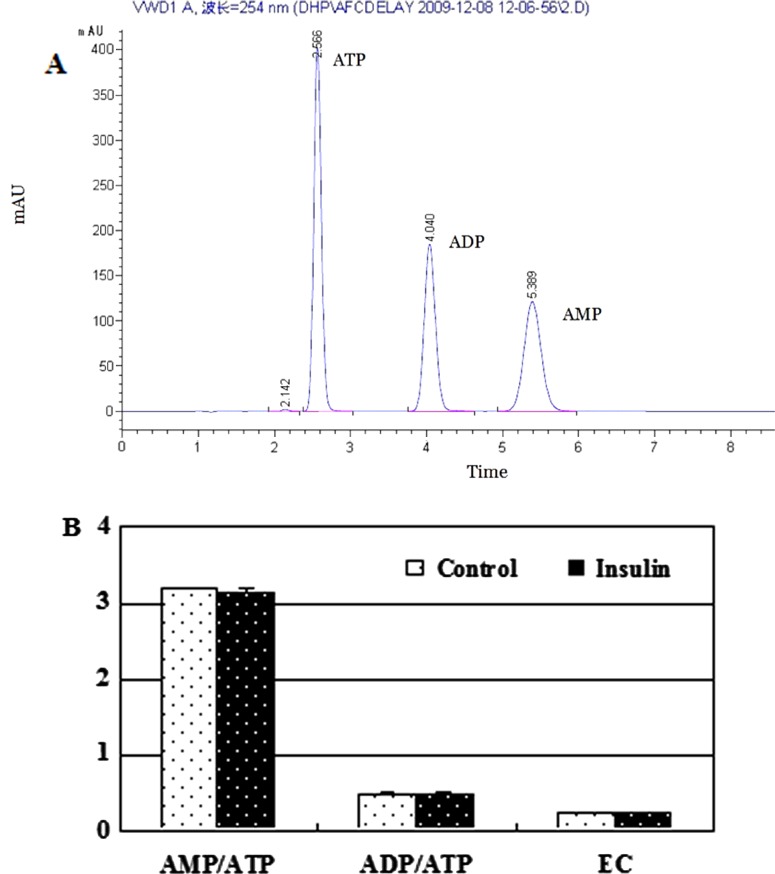
Insulin has no influece on AMP–ATP ratio, ADP–ATP ratio and EC Effects of insulin exerted no influence on AMP–ATP ratio, ADP–ATP ratio and EC 1 h after the treatment. The differentiated L6 myotubes were treated with insulin (100 nM) for 1 h. The nts were extracted from the treated L6 myotubes and determined with HPLC. (**A**) The order of eluted nts was ATP (2.5 min), ADP (4 min) and AMP (5.3 min). (**B**) AMP–ATP ratio, ADP–ATP ratio and EC are expressed as means ± S.D. (*n*=2).

## DISCUSSION

The molecular mechanism of the effect of insulin on AMPK signalling pathways has been previously elucidated as direct phosphorylation of AMPK by PKB/Akt on Ser^485/491^, which can prevent subsequent activation of AMPK at Thr^172^ by LKB1 [13]. In addition, it has been shown that atrogin-1/MAFbx and MuRF1 are down-regulated as a result of AKT (protein kinase B)-mediated inactivation of Foxo4. This activation of AKT was shown to be mediated through the insulin-like growth factor-1 (IGF-1) receptor signalling [14]. Finally, it has been shown that activation of AMPK stimulates myofibrillar protein degradation through the expression of atrogin-1/MAFbx and MuRF1 by increasing Foxo transcription factors in skeletal muscles [15]. Hence, it could be hypothesized that insulin inhibition of MAFbx and MuRF1 is through inhibition of AMPK activation. Our results suggest that insulin/IGF-1’s inhibition of MAFbx and MURF1 might be occurring through inhibition of AMPK; however, it needs to be determined whether the inhibitory effects of insulin on the expression of MAFbx and MuRF1 through Akt-inhibited Foxo are either stronger or weaker than the inhibitory effects of insulin through AMPK inhibition.

Regulation of muscle mass depends on an intricate homoeostatic balance between muscle protein synthesis and degradation. Whereas phosphorylation-mediated activation of AMPK in skeletal muscle has been shown to down-regulate protein synthesis via inhibition of mTOR kinase, a key mediator of muscle protein synthesis [16]. AMPK has been shown to enhance muscle protein degradation through promoting the expression of two muscle-specific ubiquitin ligases, MAFbx and MuRF1 [17]. Muscle atrophy in many conditions share a common mechanism in the up-regulation of MAFbx and MuRF1, both of which are part of the ubiquitin proteasome pathway utilized for protein degradation during muscle atrophy [14].

The present study also provides experimental evidence that contributes to a better understanding of the links between cellular energy metabolism and the regulation of protein degradation by using an *in vitro* study of L6 myotubes. According to our results and others [18], insulin inhibits protein degradation. Inhibition of protein degradation in skeletal muscle might result in a decrease in free amino acids, which are substrates for ATP synthesis. Meanwhile, the inhibition of AMPK also results in less ATP production, which may constitute another factor that plays a role in down-regulating protein degradation, as the latter is an energy consuming process. Thus, insulin induces two highly related and co-ordinated metabolic processes: inhibition of the activity of the energy sensor and inhibition of protein degradation. Thus, it seems that insulin exerts these two activities in order to co-ordinate a balanced metabolism.

We used an AICAR [19] and inhibitor (compound C) [20] to determine the effects of AMPK on the mRNA expression of ubiquitin E3 ligases. When AICAR activates AMPK, we show that the mRNA expression of ubiquitin E3 ligases is enhanced, which is consistent with a previous study [6]. As compound C is widely used as an AMPK inhibitor, we treated myotubes with compound C to inhibit AMPK. Our results show that pre-treatment with an AMPK inhibitor attenuate the AICAR-induced increase in mRNA expression of MuRF1 and MAFbx.

Insulin inhibited AMPK, showing a reduced phosphorylation of ACC in myocardium [21]. In myocardium, the decreased phosphorylation of AMPK at Thr^172^ may occur in an Akt-dependent manner [22]. Insulin also inhibits AMPK in 3T3-L1 adipocytes [23]. In the present study, we conclude that insulin reduces the phosphorylation and inhibits AMPK in L6 myotubes. Furthermore, the effects of this AMPK inhibition depend on the activation of Akt. Insulin significantly down-regulated the expression of MAFbx and MuRF1. Furthermore, activated AMPK induces the expression of MAFbx and MuRF1. This implies that insulin might inhibit the expression of ubiquitin E3 ligases through inhibition of AMPK.

Of note, the observation for the acute changes of AMPK activity (via AMPK and ACC phosphorylation in 30 min) and the observation for the prolonged effects (mRNA level, 24 h) do not necessarily provide the linear conformity to regulations by either insulin or AMPK pathway respectively. However, it has been previously shown that in cardiomyocytes, either acute or chronic (up to 7 days) AMPK activation increased MAFbx and MuRF1 mRNA levels in the heart *in vivo* [24]. Hence, it can be rationalized that a similar mechanism is operant in the myotubes.

The α subunit is the catalytic subunit of AMPK and includes the α1 and α2 sub-types. It is interesting that AMPK α2 is highly expressed in heart, liver and skeletal muscles, whereas AMPK α1 is ubiquitously distributed. In skeletal muscles, the subtype α2 constitutes 80% of the total AMPKα subunit [25]. This implies that it plays an important metabolic role in skeletal muscles. The two sub-types of AMPK α have different functions. For example, AMPK α1, but not α2, takes part in regulation of glucose uptake and fatty acid oxidation [26–28]. In the present study, it was shown that insulin down-regulated AMPK α2 mRNA, but had no effects on that of AMPK α1. In regard to regulating ubiquitin E3 ligases, it is our assumption that AMPK α2 is involved in the process. We found that insulin reduced AMPK α2 mRNA, which was in line with reduced expression of ubiquitin E3 ligases. Figure 4 showed that, although it was not statistically significant, insulin slightly induced AMPK α1 mRNA. This is consistent with this sub-type playing a role in regulating glucose uptake [26]. AMPK is a sensor of cellular energy. Energy status and AMP–ATP ratio regulate the phosphorylation and activity of AMPK in skeletal muscles. Thus, we measured the AMP–ATP ratio, ADP–ATP ratio and EC in L6 myotubes. We found that insulin did not affect AMP–ATP ratio, ADP–ATP ratio or EC. These data indicate that insulin inhibits AMPK without affecting the energy status of myotubes.

In conclusion, insulin, as an anti-catabolic hormone, may antagonize the expression of ubiquitin E3 ligases, thus serving as a key enzyme in protein degradation by inhibiting AMPK activity in an Akt-dependent manner in L6 myotubes. Furthermore, insulin reduces AMPK α2 mRNA levels, which may also take part in inhibiting the activation of AMPK.

## Abbreviations

ACC: acetyl CoA carboxylase
AICAR: 5-aminoimidazole-4-carboxamide-1-β-4-ribofuranoside
Akt/PKB: protein kinase B
AMPK: AMP-activated protein kinase
EC: energy charge
Foxo: forkhead box O
GAPDH: glyceraldehyde-3-phosphate dehydrogenase
IGF-1: insulin-like growth factor-1
MAFbx: muscle atrophy F-box
mTOR: mammalian target of rapamycin
MuRF1: muscle RING finger 1
p-ACC: phosphor-ACC
p-AMPK: phosphor-AMPK
qRT-PCR: quantitative real time PCR
TSC2: tuberous sclerosis protein-2
